# 

**Published:** 1900-04-28

**Authors:** 


### DEATH.

Nash.—On the 6th inst., at St. Bartholomew's Hospital, Sarah Lees (Cissie), eldest daughter of the late Charles Barnes Nash, aged 36 years, after six months' illness. (693)

### NOTICE TO CORRESPONDENTS.

All MS., Letters, Books for Review, and other matters intended for the Editor should be addressed The Editor, "The Hospital," The Hospital Building, 28 & 29, Southampton Street, Strand, W.C.

The Editor cannot undertake to return rejected MS., even when accompanied by stamped directed envelope.

### Notice to Advertisers and Subscribers.

All Advertisements, Orders for Copies of The Hospital, and Business Communications should be addressed to **THE MANAGER (Not to the Editor)**, The Hospital, The Hospital Building, 28 & 29, Southampton Street, Strand, London, W.C.

The Cost of Subscription, post free, is as follows:—Per week, 2½d.; per quarter, 2s. 8d.; per half-year, 5s. 3d.; per annum, 10s. 6d. Foreign:—Per annum, 15s. 2d., payable, in all cases, in advance.

**NOTICE.—Vol. XXVII. of "The Hospital" (October, 1899, to April, 1900), in handsome cloth binding, will shortly be on sale at the Office of this Journal, price 7s. 6d. Cases for binding the Numbers contained in this Volume 1s. 6d. Index to Vol. XXVII., 2½d., post free.**

**The Monthly Part for May will be ready on 1st May. Price 6d., by post 8d.**

### BOOKS RECEIVED.

G. W. Bacon and Co.

"Bacon's War Atlas of South Africa."

J. and A. Churchill.

"The Physical Examination and Development of Public School Boys." By Cecil Hawkins, M.A.

H. J. Glaisher.

"The Nurse's Report Book," Compiled by "K. H."

The Scientific Press.

"Burdett's Official Nursing Directory, 1900."

Cassell and Co.

"Tuberculosis, Its Nature, Prevention, and Treatment." By Alfred Hillier, B.A., M.D., C.M.

Lawrence Greening and Co.

"America Abroad." Edited by J. W. Cundall.

Allman and Son.

"Disinfection and Disinfectants." By John Gay, M.R.C.S., D.P.H.

S. W. Partridge and Co.

"To the Men at the Front."

Bright's (Limited).

"Report of the Medical Officer of Health of the Borough of Bournemouth."

Hayman, Christy, and Lilly.

"The Nurse's Report Book." By C. M. Lohr.

Baillière, Tindall, and Cox.

"Diseases of Women and Uterine Therapeutics." By H. Macnaughton-Jones, M.D., M Ch. Eighth edition. Revised and enlarged.

### The Student of Medicine.

### APPOINTMENTS.

J. Anderson, M.D., appointed Health Officer for the Shire of Heytesbury, Victoria. W. Ayres, M.D., appointed Medical Officer for the Second Kingswinford District of the Stourbridge Union. G. H. Brand, L.R.C.P., L.M.Irel., L.S.A.Lond., appointed Honorary Medical Officer to the Northampton and County Association for the Blind. W. J. Darby, L.R.C.S.I., appointed Surgeon to H.M. Prison, Auckland, New Zealand. W. d'E. Emery, M.D., B.Sc.Lond., M.R.C.S., appointed Lecturer on Hygiene to the Birmingham and Midland Institute. D. Ferrier, M.B., Ch.B.Edin., appointed Resident Physician to the Edinburgh Royal Infirmary. T. C. Fisher, M.D., appointed Government Medical Officer and Vaccinator at Bowral, New South Wales. M. B. Foster, M.R.C.S., L.R.C.P.Lond., appointed Medical Officer for the First District of the Ware Union. W. B. Gow, M.D., appointed Medical Superintendent of the Lunatic Asylum, Wellington, N.Z. A. H. Hayes, M.R.C.S., L.R.C.P., appointed Medical Officer to the Casualty Department at the East London Hospital for Children, Shadwell, E. H. C. Holden, L.S.A., appointed Assistant Medical Officer to the Woolwich Union Infirmary. H. F. Kingston, M.B.Dubl., appointed Medical Officer for the Alsager and Odd Rode District of the Congleton Union. A. Kinsey-Morgan, M.D.Durh., M.R.C.S.Eng., appointed Coroner for the County Borough of Bournemouth. H. Knight, M.R.C.S., L.R.C.P Lond., appointed Medical Officer for the Third District of South Stoneham Union. S. T. Lord, M.R.C.S., L.R.C.P., appointed Medical Officer of Health to the Castleton Urban District Council. J. G. Macmillan, M.B., appointed Health Officer for the Shire of Wimmera, Victoria. J. M. Martin, M.B., D.P.H.Camb., appointed Medical Officer of Health to the Stroud Rural District Council. S. V. Pearson, B.A., B.C.Camb., appointed House Surgeon to the East London Hospital for Children, Shadwell, E. J. Phillips, M.R.C.S.Edin., L.R.C.P.Lond., appointed Senior House Surgeon to the Bradford Royal Infirmary. R. H. Schlink, M.D., appointed Health Officer for the Shire of Wodonga, Victoria. C. H. Sills, M.R.C.S., L.R.C.P., appointed Medical Officer for the First District of the East Ashford Union. C. N. Simons, L.R.C.S.I., L.S.A., appointed Government Medical Officer and Vaccinator at Campbelltown, New South Wales. F. L. Titley, M.R.C.S., L.R.C.P., appointed Medical Officer for the Swinefleet District of the Goole Union. G. G. Vatve, M.D., appointed Assistant Darbar Surgeon to the Albert Edward Hospital, Kolhapur. J. Wassell, M.B., appointed Assistant Health Officer at Brisbane. J. E. Wolfhagen, M.B., C.M Edin., appointed Honorary Medical Officer to the Hobart General Hospital.

Hovis

(TRADE MARK.)

An exceptionally nutritious and digestive Bread, especially suitable for the dyspeptic and convalescent: It is both tasty and appetising.

Agents' addresses will be sent on application to The Hovis-Bread Flour Co. Ld., Macclesfield.

Accouchement Requisites.

Ambulance Cases.

### NOW READY,

A REPRINT OF

### A NURSING HANDBOOK

TOGETHER WITH S. MAW, SON, AND THOMPSON'S

### COMPLETE CATALOGUE OF NURSING REQUISITES.

The above work is now republished at a cost of 1s. per copy. Messrs. S. M., S., and T. have been compelled to make this charge on account of the extreme popularity of the work and the great expense incurred in its production.

***Post Free on receipt of 1s.***

### S. MAW, SON, and THOMPSON,

7 to 12, ALDERSGATE STREET, LONDON, E.C.

